# Characterization of Fiber-Optic Vector Magnetic Field Sensors Based on the Magneto-Strictive Effect

**DOI:** 10.3390/s23167127

**Published:** 2023-08-11

**Authors:** Ning Li, Yuren Chen, Chaofan Zhang, Jie Nong, Wenjie Xu, Zhencheng Wang, Junbo Yang, Yang Yu, Zhenrong Zhang

**Affiliations:** 1 Guangxi Key Laboratory of Multimedia Communications and Network Technology, School of Computer, Electronic and Information, Guangxi University, Nanning 530004, China; 2113391102@st.gxu.edu.cn (N.L.); 2113391088@st.gxu.edu.cn (Y.C.); 2113391106@st.gxu.edu.cn (J.N.); 2113391116@st.gxu.edu.cn (W.X.); 2112401015@st.gxu.edu.cn (Z.W.); 2 College of Advanced Interdisciplinary Studies, National University of Defense Technology, Changsha 410073, China; c.zhang@nudt.edu.cn; 3 College of Sciences, National University of Defense Technology, Changsha 410073, China; yangjunbo@nudt.edu.cn (J.Y.); yuyang08a@nudt.edu.cn (Y.Y.)

**Keywords:** Terfenol-D (TbDyFe alloy), magneto-strictive effect, sensitivity matrix, 3D vector sensor

## Abstract

Fiber-optic magnetic field sensors have garnered considerable attention in the field of marine monitoring due to their compact size, robust anti-electromagnetic interference capabilities, corrosion resistance, high sensitivity, ease of multiplexing and integration, and potential for large-scale sensing networks. To enable the detection of marine magnetic field vector information, we propose an optical fiber vector magnetic field sensor that integrates three single-axis sensors in an orthogonal configuration. Theoretical analysis and experimental verification are conducted to investigate its magnetic field and temperature sensing characteristics, and a sensitivity matrix is established to address the cross-sensitivity between the magnetic field and temperature; experimental tests were conducted to assess the vector response of the three-dimensional (3D) vector sensor across the three orthogonal axes; the obtained experimental results illustrate the commendable magnetic field vector response exhibited by the sensor in the orthogonal axes, enabling precise demodulation of vector magnetic field information. This sensor presents several advantages, including cost-effectiveness, easy integration, and reliability vectorially. Consequently, it holds immense potential for critical applications in marine magnetic field network detection.

## 1. Introduction

Due to the special environmental and physical characteristics of the ocean, magnetic fields are one of the few signal carriers that can be transmitted over long distances in the ocean. Magnetic fields represent vector fields that encompass both magnitude and directional information and are typically described by three independent magnetic field components [1]. Acquiring marine magnetic field information plays a pivotal role in supporting various technical aspects, such as marine mineral resource exploration [2], underwater magnetic target detection [3], and autonomous navigation of underwater submersibles [4]. The demand for high-performance, miniaturized triaxial magnetic sensors has grown significantly to address emerging applications requiring weak magnetic sensing capabilities. Utilizing three-axis magnetic sensors to measure the three-component magnetic information allows for the extraction of richer signal features, catering to the detection requirements of underwater magnetic field arraying and enabling low-cost large-area monitoring capabilities. In light of this, the accurate acquisition of ocean magnetic field information holds paramount importance, particularly in applications related to foreign intrusion submarine early warning, underwater miniature reconnaissance robot intrusion monitoring, torpedo detection, and underwater stealth missile countermeasures. These areas of research and development necessitate cutting-edge sensing technologies to enhance the capabilities of underwater systems and ensure effective monitoring and defense.

Fiber-optic magnetic field sensors have garnered significant attention within the realm of marine monitoring applications due to their compact size, robust resistance to electromagnetic interference, exceptional corrosion resistance, high sensitivity, effortless multiplexing and integration capabilities, and their ability to facilitate large-scale sensing networks. Leveraging the dielectric properties of optical fibers, the integration of magnetically sensitive materials into fiber-optic magnetic field sensors represents a promising avenue for advancement. Notably, researchers have recently proposed diverse types of fiber-optic magnetic field sensors, primarily focusing on magneto-fluid (MF) [5,6,7], magneto-optical materials (MO) [8,9], and giant magneto-strictive materials (GMM) [10,11,12,13]. Given the distinct advantages associated with different sensing structures, a combined approach enables exploiting the full potential of the integrated sensing system. By synergistically harnessing the strengths of these diverse methods, the resulting sensing system achieves optimal performance and functionality.

For instance, Ji et al. proposed a fiber-optic Fabry–Perot cavity (F–P) magnetic field sensor where an MF is injected into the F–P cavity as the magnetic field measurement unit. To compensate for temperature effects, a fiber Bragg grating (FBG) is incorporated at the fiber insertion end of the F–P cavity. Experimental results reveal a sensitivity of 0.53 nm/mT for magnetic field measurements within the range of 20–60 mT, with a resolution reaching 37.7 µT [14]. In 2018, Dong et al. introduced a magnetic field sensor based on a magnetic fluid permeable phase-shifted fiber grating consisting of a fiber grating with a micrometer gap between two segments. The refractive index of the magnetic fluid varies in response to the magnetic field, influencing the transmission spectrum as it enters the sensing structure through the gap. The sensitivity of the magnetic field strength, ranging from 0 to 12 mT [15], is measured at 24.2 pm/mT. It is worth noting that magnetic fluid materials possess a naturally volatile nature, making them susceptible to magnetic field saturation. Magnetic field sensors utilizing the refractive index effect of magnetic fluids, as discussed above, are significantly influenced by the properties of the fluids. As a consequence, their sensing performance exhibits a limited dynamic range for detecting magnetic fields and poor long-term stability. Their practical application in monitoring magnetic field information over long distances is constrained. To address these limitations, alternative approaches are required to ensure extended monitoring capabilities and enhance the sensor’s performance in real-world environments.

Optical fiber magnetic field sensors utilizing MO materials operate based on the Faraday spin effect, enabling magnetic field measurements. However, the sensor sensitivity is constrained by the Verdet constant. To overcome this limitation, Sun et al. employed a piece of terbium-doped silicate fiber, enabling direct magnetic field measurement by detecting the polarization rotation induced by the Faraday effect. This approach compensates for the low Verdet constant of the fiber [16]. In 2020, Jiang et al. introduced a novel fiber-optic magnetic field sensor based on yttrium iron garnet (YIG) [17]. The sensor underwent testing in liquid nitrogen, demonstrating a measurement capability of up to 24.7 mT at 77 K. Nonetheless, the magnetic field sensor relying on magneto-optical materials encounters challenges such as a complex system optical path, costly materials, and low robustness. Additionally, the polarization state of the fiber is easily disturbed in practical application environments. The temperature crossover effect poses a significant obstacle to the practical deployment of Faraday rotation-based fiber-optic magnetic field sensors.

The remarkable responsiveness and stability exhibited by GMM, such as Fe–Ga alloy or Terfenol-D, in response to magnetic fields have sparked new possibilities in the development of fiber-optic magnetic field sensors. These sensors, based on GMM, are designed to leverage the variations in the properties of the GMM induced by the ambient magnetic field and translate them into wavelength changes in the fiber. In this design, FBG serves as a dependable and practical passive device capable of not only sensing the external environment to facilitate measurements of stress, temperature, and other parameters but also enabling easy multiplexing and the realization of large-scale sensing networks.

Numerous research endeavors have investigated magnetic field measurements utilizing sensors integrated with Terfenol-D and FBG. For instance, Yang et al. employed a magnetron sputtering method to deposit TbDyFe films on the etched side circles of FBGs, achieving a magnetic field sensitivity of 0.9 pm/mT within the 0–50 mT range [10]. In 2013, Dai et al. proposed a magnetic field sensor based on a spiral microstructured fiber grating coated with Terfenol-D. By utilizing a femtosecond laser processing system to inscribe a helical microstructure into the FBG cladding, the sensitivity of this magnetic field sensor was enhanced by approximately five times compared to that of the non-helical-structured FBG. The sensitivity reached approximately 0.7 pm/mT within the magnetic field strength range of 0–140 mT [11]. However, due to the expensive nature of femtosecond laser equipment, the fabrication of this sensor incurs high costs, rendering it unsuitable for large-scale sensing applications. In 2016, García-Miquel et al. explored temperature and strain monitoring utilizing Terfenol-D in conjunction with FBGs. Their design entailed three FBG detection branches and a complex sensor structure, necessitating strict orthogonality between two of the FBGs, thereby increasing the difficulty of practical measurements [12]. In 2021, Zhan et al. immobilized two identical FBGs onto Terfenol-D to monitor magnetic field strength, achieving a maximum sensitivity of 0.87 pm/mT within the range of 8−28 mT. While the sensor facilitated temperature-insensitive magnetic field measurements, it was unable to monitor the vectorial properties of the magnetic field [13]. Despite the high sensitivity demonstrated by some of the aforementioned fiber-optic magnetic field sensors in spectral drift intensity measurements, they still exhibit limitations in vector magnetic field testing, multiplexing, and fabrication complexity.

In this study, we present a novel approach to address the limitations observed in existing electrical fiber-optic magnetic field sensors, including weak anti-electromagnetic interference, large size, power supply requirements, networking complexities, and the inability to operate effectively in long-term underwater conditions. To overcome these challenges, we propose an orthogonally integrated fiber-optic vector magnetic field sensor comprising three single-axis sensors. The designed three-dimensional vector sensor exhibits remarkable sensitivity to magnetic field and temperature, thereby facilitating long-distance detection and temperature compensation. Experimental results demonstrate the sensor’s excellent magnetic field vector response across the three orthogonal axes, enabling accurate demodulation of vector magnetic field information. Our primary focus is on evaluating the fabrication process to develop stable, reliable, and multiplexable vector fiber-optic magnetic field sensors suitable for marine magnetic field information detection applications.

## 2. All-Fiber-Optic Magnetic Field Sensor

### 2.1. Sensor Design and Fabrication

**Figure 1:**
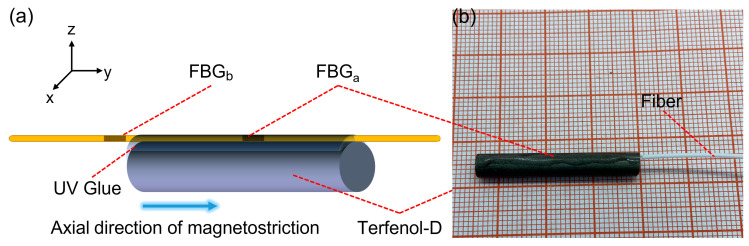
(**a**) Schematic diagram of the sensor structure; (**b**) Physical picture of the sensor.

The schematic diagram of the designed magneto-strictive effect based all-fiber FBG magnetic field sensor structure is illustrated in Figure 1a. The sensor comprises a Terfenol-D rod with dimensions of Φ5 mm × 30 mm and a customized cascaded fiber Bragg short-period grating (FBG) with central reflection wave peaks at 1550.034 nm and 1555.003 nm, respectively. The Terfenol-D rod is horizontally fixed on an optical damping platform, while the fiber grating area is horizontally attached to the upper surface of the Terfenol-D rod using a three-dimensional adjustment frame. To ensure a secure connection between the Terfenol-D rod and the grating, a certain amount of prestress is applied to one end of the fiber through the displacement platform. The fiber Bragg grating attached to the Terfenol-D material is denoted as ${\mathrm{FBG}}_{a}$, representing the reflection center peak labeled as $peak_{a}$. Additionally, a cascaded grating denoted as ${\mathrm{FBG}}_{b}$, corresponding to the reflection center peak labeled as $peak_{b}$, is integrated into the tail fiber of the sensor, as depicted in Figure 1a. Moreover, the surfaces of the grating are uniformly coated with UV adhesive along the axial direction of the Terfenol-D bar to facilitate proper bonding. Subsequently, the UV adhesive is cured by exposing it to a UV lamp for 60 s. The physical appearance of the all-fiber FBG magnetic field sensor sample is presented in Figure 1b.

### 2.2. Theoretical Analysis

Terfenol-D has a high magneto-strictive coefficient. When subjected to an applied magnetic field H, the Terfenol-D generates an axial stress ε within its linear region, which can be mathematically represented as [18]:

(1) $$\varepsilon =\frac{\Delta L}{L}=C_{f}H,$$

where L, ΔL, and $C_{f}$ denote the initial length, deformation of the Terfenol-D, and magneto-strictive coefficient, respectively. Consequently, variations in the applied magnetic field lead to changes in the axial stress ε induced by the elongation of the super magneto-strictive material, which in turn affects the wavelength shift of the center of the FBG reflection spectrum. Assuming a constant temperature, the offset resulting from the influence of the axial stress ε can be expressed as:

(2) $$\Delta \lambda_{B}{}_{\left(\varepsilon \right)}=(1-p_{e})\varepsilon \lambda_{B},$$

where $p_{e}$ represents the effective elastic optical coefficient of the fiber and ε corresponds to the axial stress experienced by the FBG. For a typical quartz single-mode fiber, $p_{e}$ is approximately 0.22 [19]. Furthermore, it is important to consider the effects of temperature variations, which can result in wavelength drift. Consequently, when the FBG experiences temperature changes, the offset arising from the combined thermal expansion effect and thermo-optical effect can be represented by Equation (3). Here, α and ξ represent the thermal expansion coefficient and thermo-optical coefficient of the grating, respectively.

(3) $$\Delta \lambda_{B}{}_{\left(T\right)}=(\alpha +\xi )\Delta T\lambda_{B},$$

According to Equations (1)–(3), the relationship between the observed changes in the FBG, influenced by both magnetic field and temperature within the linear range, and the magnetic field under measurement can be measured as follows:

(4) $$\Delta \lambda_{B}=\Delta \lambda_{B}{}_{\left(\varepsilon \right)}+\Delta \lambda_{B}{}_{\left(T\right)}=\left(1-p_{e}\right)C_{f}H\lambda_{B}+(\alpha +\xi )\Delta T\lambda_{B},$$

It is evident that the wavelength shift of the central point of the FBG reflection spectrum exhibits a linear correlation with both variations in magnetic field and temperature. Although temperature changes are independent of the magnetic field, they still influence the occurrence of the offset as an environmental factor. If the impact of temperature can be disregarded, Equation (4) can be simplified as:

(5) $$\frac{\Delta \lambda_{B}}{H}=\left(1-p_{e}\right)C_{f}\lambda_{B},$$

Hence, the sensor presented in this study incorporates temperature compensation techniques to mitigate the impact of ambient temperature on magnetic field measurements, ensuring that the output signal of the sensor is solely associated with variations in magnetic field intensity. Based on the aforementioned analysis, it is evident that the alteration of external environmental parameters can be indirectly detected by monitoring the drift of the central wavelength peak of the FBG reflection spectrum.

### 2.3. Magnetic Field Response Test

**Figure 2:**
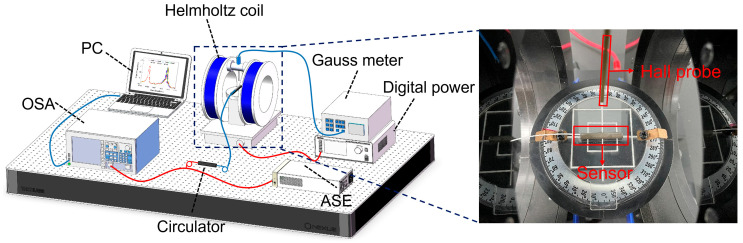
Schematic diagram of the magnetic field test system, illustrating the placement of the sensor within the Helmholtz coil.

The experimental configuration of the proposed magnetic field sensor is depicted in Figure 2. A high-precision digital power supply (CH-F2030, −10 A~10 A) is employed to drive a one-dimensional Helmholtz coil (CHY12-500, −50 mT~50 mT), enabling the generation of a stable magnetic field by adjusting the output current. The magnetic field intensity is accurately measured using a Gauss meter (CH-1800, 0~30 T). An amplified spontaneous emission light source (ASE, 1520~1620 nm) serves as the input light source, which is transmitted to the magnetic field sensor through a circulator connected to the grating via a pigtail. The reflected light is then directed through the circulator into the spectrometer (OSA-AQ6370D, 600~1700 nm), where real-time wavelength offsets can be observed. The obtained spectral data is subsequently transferred to a personal computer (PC) for further analysis and processing. The magnetic field sensor sample consists of a cascaded grating with Bragg reflection peaks at 1550.034 nm and 1555.003 nm, in conjunction with the Terfenol-D. The sensor sample is positioned at the center of the one-dimensional Helmholtz coil, with the magnetic field direction aligned parallel to the magnetostriction direction of Terfenol-D, as illustrated in Figure 2. The magnetic field sensor sample consists of a cascade grating with Bragg reflection peaks of 1550.034 nm and 1555.003 nm and Terfenol-D. The sensor sample is placed into the center of a one-dimensional Helmholtz coil with the magnetic field direction parallel to the magnetostriction direction of Terfenol-D, as shown in the illustration in Figure 2.

**Figure 3:**
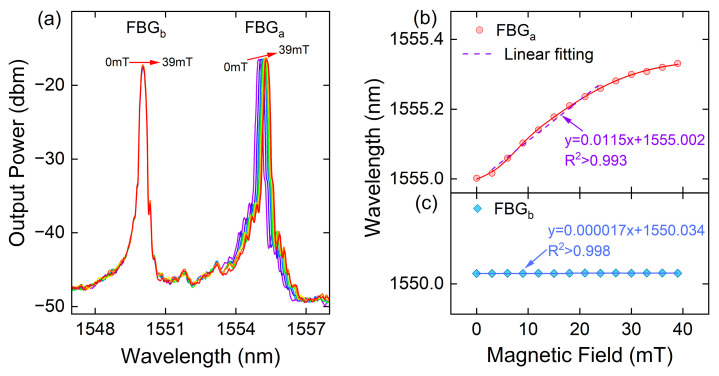
(**a**) The reflection spectra and fitted curves of the sensor under different magnetic fields; The wavelength shift of (**b**) ${\mathrm{FBG}}_{a}$ and (**c**) ${\mathrm{FBG}}_{b}$ when increasing the applied magnetic field from 0 to 39 mT.

In the magnetic field response test, to prevent the coil from heating up due to energization, the Helmholtz coil is equipped with a water-cooled circulation pump to keep the temperature around the sensing structure at room temperature. The DC magnetic field strength generated by the Helmholtz coil was initially calibrated using a Hall probe (HCHD801F) to ensure accuracy. Subsequently, the magnetic field response test was conducted under controlled conditions at a temperature of 24.3 °C. The magnetic field strength was incrementally increased from 0 mT to 39 mT with a gradient of 3 mT. Experimental data were recorded at intervals of 3 min to obtain a stable output waveform. Figure 3a depicts the FBG reflection spectra of the sensor at different magnetic field strengths. It is observed that with the increase in magnetic field strength, $peak_{a}$ exhibits a redshift, while $peak_{b}$ remains unchanged. Figure 3b presents the nonlinear fitted curve of the FBG reflection center waveform. This analysis confirms that the sensor demonstrates a linear response within the range of 3−27 mT. The magnetic field sensitivity of the magnetic field sensor proposed in this paper is defined as [6]:

(6) $$\left\{\begin{array}{l} S_{H-peak_{a}}=\frac{\Delta \lambda_{peak_{a}}}{\Delta H}\\ S_{H-peak_{b}}=\frac{\Delta \lambda_{peak_{b}}}{\Delta H} \end{array}\right.,$$

The magnetic field sensitivity of the proposed FBG cascade sensor ${\mathrm{FBG}}_{a}$(${\mathrm{FBG}}_{b}$) is denoted as $S_{H-peak_{a}}$($S_{H-peak_{b}}$), where $S_{H-peak_{a}}$ corresponds to the sensitivity of ${\mathrm{FBG}}_{a}$ and $S_{H-peak_{b}}$ corresponds to the sensitivity of ${\mathrm{FBG}}_{b}$. The peak drifts of ${\mathrm{FBG}}_{a}$ and ${\mathrm{FBG}}_{b}$ are represented by $\Delta \lambda_{peak_{a}}$ and $\Delta \lambda_{peak_{b}}$, respectively, while ΔH denotes the change in magnetic field intensity. In Figure 3b, it can be observed that as the magnetic field increases from 3 mT to 27 mT, resulting in a wavelength shift of $peak_{a}$ from 1555.016 nm to 1555.291 nm, the peak drift Δλ is measured to be 0.275 nm. The linear fitting curve of $peak_{a}$ exhibits a high correlation coefficient of $R^{2}=0.993$. Based on these observations, the magnetic field sensitivity of the proposed sensor is determined as $S_{H-peak1}=11.5\;\mathrm{pm}/\mathrm{mT}$. Figure 3c demonstrates that the linear slope of the fitted curve for ${\mathrm{FBG}}_{b}$ is nearly zero, indicating it does not respond to the magnetic field.

### 2.4. Temperature Response Test

**Figure 4:**
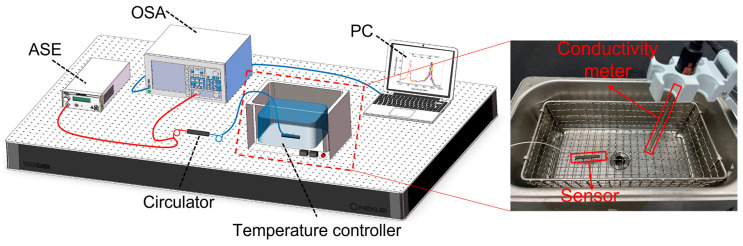
Schematic diagram of the temperature experiment system.

The theoretical analysis reveals that the fiber-optic magnetic field sensor utilizing the FBG magneto-strictive effect possesses dual functionality by being responsive to magnetic fields and capable of temperature sensing. In this study, a magnetic field sensor employing a cascaded grating temperature compensation approach with temperature-insensitive characteristics is proposed. To assess the temperature response, a similar experimental optical setup as the one used for magnetic field response is employed. The experimental system, illustrated in Figure 4, comprises an amplified self-radiating broadband light source (ASE), a spectrometer (OSA), a conductivity meter (WSA1521), a temperature controller (KQ2200DE), and a signal processing computer (PC), along with a water tank with a capacity of approximately 2 L.

**Figure 5:**
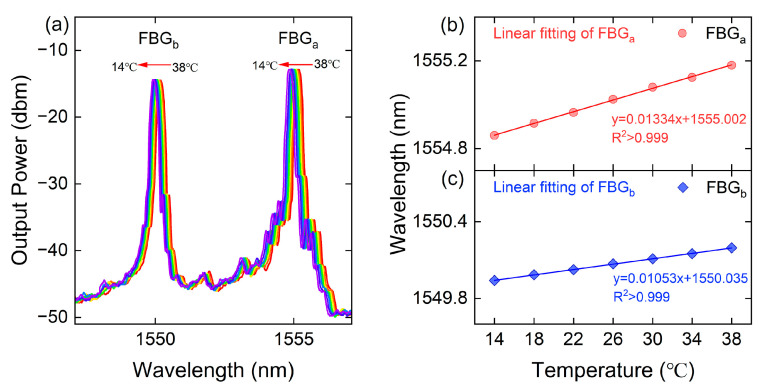
(**a**) The reflection spectra and fitted curves of the sensor under different temperatures. The wavelength shift of (**b**) ${\mathrm{FBG}}_{a}$ and (**c**) ${\mathrm{FBG}}_{b}$ with reducing temperature from 38 to 14 °C.

As shown in the inset of Figure 4b, this illustrates the experimental setup in which the cascaded sensor sample was immersed in a temperature-controlled water tank. The sample underwent a gradual heating process until reaching a temperature of 38 °C, which was maintained for a duration of ten minutes. When the temperature inside the sensor was the same as that in the temperature tank (no drift of the central reflection peak of the OSA output reflection spectrum), the recording of the output spectrum commenced during the natural cooling phase. The obtained FBG reflection spectra of the sensor at various temperatures are presented in Figure 5a. It is evident that both $peak_{a}$ and $peak_{b}$ experience a blue shift as the temperature increases. Similarly, the temperature sensitivity of the proposed magnetic field sensor, as described in this study, can be defined by:

(7) $$\left\{\begin{array}{l} S_{T-peak_{a}}=\frac{\Delta \lambda_{peak_{a}}}{\Delta T}\\ S_{T-peak_{b}}=\frac{\Delta \lambda_{peak_{b}}}{\Delta T} \end{array}\right.,$$

The temperature sensitivity of the proposed FBG cascade sensor, denoted as $S_{T-peak_{a}}$($S_{T-peak_{b}}$), represents the responsiveness of ${\mathrm{FBG}}_{a}$(${\mathrm{FBG}}_{b}$) to temperature variations. Meanwhile, $\Delta \lambda_{peak_{a}}$($\Delta \lambda_{peak_{b}}$) indicates the respective peak drifts observed, and ΔT signifies the magnitude of the temperature variation. Figure 5b,c display the linear fitting curves illustrating the sensitivity of temperature associated with the wavelengths of the two FBG reflection centers. The temperature sensitivities of the cascaded grating sensors are quantified as $S_{T-peak1}=13.3\;\mathrm{pm}/\mathrm{mT}$ and $S_{T-peak2}=10.5\;\mathrm{pm}/\mathrm{mT}$, as depicted in the figures.

By considering the aforementioned experimental outcomes regarding the magnetic and temperature responses of cascaded gratings, it is possible to establish a sensitivity matrix. This matrix integrates the magnetic field sensitivity and the temperature sensitivity associated with the wavelength of the FBG reflection center. The primary objective is to enable dual parametric sensing, encompassing both magnetic field and temperature measurements.

(8) $$\left[\begin{array}{l} \Delta \lambda_{{\mathrm{peak}}_{a}}\\ \Delta \lambda_{{\mathrm{peak}}_{b}} \end{array}\right]=\left[\begin{array}{l} S_{H-{\mathrm{peak}}_{a}}\\ S_{H-{\mathrm{peak}}_{b}} \end{array}\right.\left.\begin{array}{l} S_{T-{\mathrm{peak}}_{a}}\\ S_{T-{\mathrm{peak}}_{b}} \end{array}\right]\left[\begin{array}{l} \Delta H\\ \Delta T \end{array}\right]=K\left[\begin{array}{l} \Delta H\\ \Delta T \end{array}\right],$$

Equation (8) introduces the key variables and coefficients involved in the matrix. The wavelength drifts of the FBG reflection center wavelengths, represented by $\Delta \lambda_{peak_{a}}$ and $\Delta \lambda_{peak_{b}}$, are utilized. Additionally, ΔH and ΔT correspond to the variations in the magnetic field and temperature, respectively. The coefficient K signifies the sensitivity matrix coefficient. Furthermore, $S_{H-peak_{a}}$ and $S_{H-peak_{b}}$ represent the magnetic field sensitivity of the sensor within the linear region of the Bragg reflection characteristic wave peak. Similarly, $S_{T-peak_{a}}$ and $S_{T-peak_{b}}$ indicate the temperature sensitivity of the sensor. Consequently, Equation (8) can be expressed as:

(9) $$\left[\begin{array}{l} \Delta H\\ \Delta T \end{array}\right]=\frac{1}{\det(K)}\left[\begin{array}{l} S_{T-{\mathrm{peak}}_{b}}\\ -S_{H-{\mathrm{peak}}_{b}} \end{array}\right.\left.\begin{array}{l} -S_{T-{\mathrm{peak}}_{a}}\\ S_{H-{\mathrm{peak}}_{a}} \end{array}\right]\left[\begin{array}{l} \Delta \lambda_{{\mathrm{peak}}_{a}}\\ \Delta \lambda_{{\mathrm{peak}}_{b}} \end{array}\right],$$

where the coefficient K is determined as $K=S_{H-peak_{a}}*S_{T-peak_{b}}-S_{H-peak_{b}}*S_{T-peak_{a}}$. Subsequently, the respective magnetic field sensitivity and temperature sensitivity are incorporated into the aforementioned equation to yield:

(10) $$\left[\begin{array}{l} \Delta H\\ \Delta T \end{array}\right]=\frac{1}{119.168}\left[\begin{array}{l} 10.5pm/℃\\ -0.0017pm/℃ \end{array}\right.\left.\begin{array}{l} -13.3pm/mT\\ 11.5pm/mT \end{array}\right]\left[\begin{array}{l} \Delta \lambda_{\mathrm{peak}1}\\ \Delta \lambda_{\mathrm{peak}2} \end{array}\right],$$

Through calculations, the condition number of the sensitivity matrix is determined to be 3.1571. The smaller value of the condition number indicates higher resistance to interference for the sensor [20]. It is worth noting that the sensor presented in this study exhibits cross-sensitivity to both magnetic field and temperature, with negligible effects from the small magnetic field and temperature variations. Additionally, the temperature characteristics of the sensor have been experimentally verified, highlighting the effectiveness of the cascaded grating-temperature compensation method in mitigating the influence of ambient temperature fluctuations on magnetic field strength measurements.

### 2.5. Two-Dimensional Vector Characteristic Test

**Figure 6:**
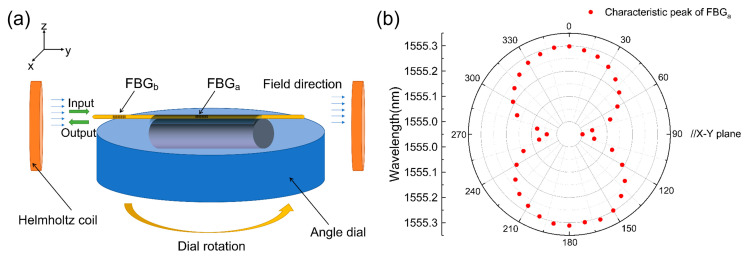
(**a**) Schematic diagram of a two-dimensional vector magnetic field detection device; (**b**) The relationship between the Bragg central reflection wave peak of the X–Y plane and magnetic field direction.

The magneto-strictive effect of the Terfenol-D is influenced not only by the strength of the magnetic field but also by the direction in which the magnetic field acts upon the Terfenol-D. To further ascertain the vectorial nature of the sensor, this subsection examines the vectoriality of the magnetic field within the two-dimensional (2D) plane, as depicted in Figure 6a. The X–Y plane of the sensor is positioned parallel to the center of the angle disc; for this purpose, the angle disc is set into rotation around the *z*-axis. It is important to note that the direction of light propagation (as indicated by the positive direction of the *y*-axis in Figure 6a) is consistently parallel to the magnetostriction direction. Upon raising the magnetic induction intensity to 30 mT and calibrating it using a Hall probe, the wavelength information of the wave peak at the center of Bragg reflection is recorded at 10° intervals from 0° to 360°.

Consequently, the relationship between the wave peak and the angle of the magnetic field vector is obtained, as illustrated in Figure 6b. Upon observing the response curve of the sensor with respect to magnetic field intensity, it becomes evident that Terfenol-D exhibits its maximum magneto-strictive effect when the magnetic field direction aligns with the magneto-strictive direction. In contrast, the magneto-strictive effect produced by the perpendicular magnetic field direction is almost negligible. Figure 6b visually demonstrates the sensor’s favorable two-dimensional vectoriality within the X–Y plane; the sensor exhibits a periodicity of 180° for vector magnetic field sensing. Notably, as the angle between the magnetic field direction and the sensor’s axial direction increases from 0° to 90°, the Bragg reflection center wave peak offset undergoes a transition from 1555.301 nm to 1555.002 nm. Conversely, when the angle shifts from 90° to 180°, the Bragg reflection center wave peak offset reverts from 1555.002 nm to 1555.311 nm, thus exhibiting symmetry. This behavior persists within the range of 180° to 360°.

## 3. Vector Magnetic Field Sensor

### 3.1. Sensor Design and Fabrication

**Figure 7:**
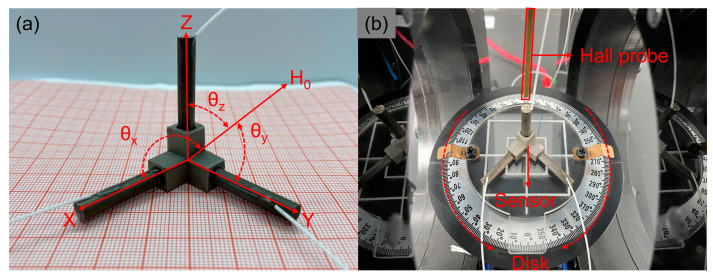
(**a**) Vector magnetic field sensor physical diagram; (**b**) Schematic diagram of the three-dimensional vector magnetic field detection device.

By leveraging the measurements of one-dimensional magnetic fields, the determination of spatial magnetic field vectors can be achieved through the establishment of a three-dimensional coordinate system employing three orthogonal single-axis sensors. In order to minimize space occupation while ensuring comprehensive measurement capabilities, a compact vector magnetic field sensor has been devised in this study. The proposed structure, depicted in Figure 7a, consists of a cube holder housing three mutually perpendicular single-axis sensors. Each sensor comprises a Terfenol-D rod and FBG. This assembly design offers notable advantages such as simplicity, reliability, compactness, and ease of installation. In an ideal scenario, the three uniaxial sensors employed for three-dimensional magnetic field measurements are mutually orthogonal. These sensors are positioned along the spatial Cartesian directions within the cube structure, thereby establishing the three coordinate sensing axes: X, Y, and Z. The magnetic field components captured by each probe are denoted as $H_{x}$, $H_{y}$, and $H_{z}$, respectively. The relationship between these magnetic field components and the overall magnetic field strength is represented by Equation (11) [21]. Here, the variables $\theta_{x}$, $\theta_{y}$, and $\theta_{z}$ correspond to the angles formed between the X, Y, and Z axes, respectively, and the measured magnetic field direction. Moreover, $H_{0}$ symbolizes the composite quantity derived from the measurements of the three single-axis magnetic fields.

(11) $$\left\{\begin{array}{l} H_{x}=H_{0}\cos(\theta_{x})\\ H_{y}=H_{0}\cos(\theta_{y})\\ H_{z}=H_{0}\cos(\theta_{z})\\ H=\sqrt{H_{x}^{2}+H_{y}^{2}+H_{z}^{2}} \end{array}\right.,$$

Figure 7b depicts the experimental setup for characterizing the vector magnetic field sensor. The sensor is positioned at the center of a disc featuring scale markings, enabling magnetic field measurements at various angles by rotating the disc. The optical fibers corresponding to the three sensing axes are connected to the three channels of a high-speed fiber grating demodulator (OSI237). This configuration facilitates the acquisition of magnetic field component values along each direction of the vector magnetic field sensor.

### 3.2. Three-Dimensional Vector Characteristic Test

**Figure 8:**
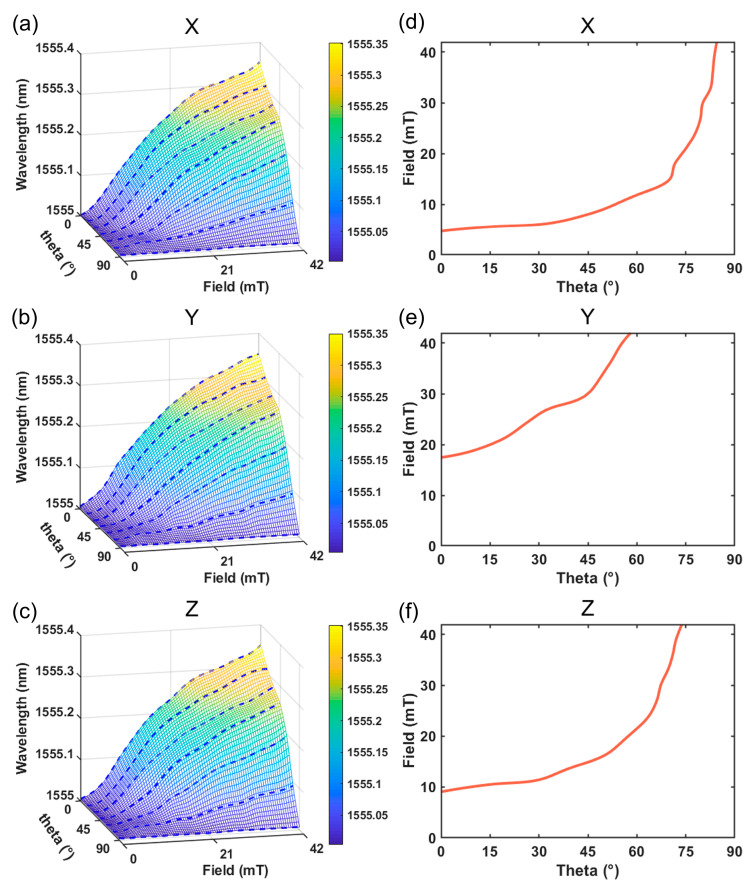
Three-dimensional mapping curves of single-axis sensors (**a**) for the *x*-axis; (**b**) for the *y*-axis; and (**c**) for the *z*-axis. Magnetic field component curves for each single-axis sensor at (**d**) the wavelength = 1555.035 nm, (**e**) at the wavelength = 1555.208 nm, and (**f**) at the wavelength = 1555.098 nm.

During the testing of vector magnetic fields, individual single-axis sensors alone are insufficient to fully characterize the comprehensive information of the vector magnetic field. This limitation arises from the close relationship between the wavelength drift of single-axis sensors and both the magnetic field strength and angle. To achieve a complete characterization of a three-dimensional vector magnetic field sensor, it is essential to establish the interdependence between the wavelength drift of each single-axis sensor and the magnetic field strength and angle. To accomplish this, the measurement of the vector magnetic field entails examining the relationship between the magnetic field and the angle of each single-axis sensor. The collected data from the high-speed fiber grating demodulator (OSI) is then transmitted to the PC for signal processing, enabling the determination of magnetic field component values along each direction of the vector magnetic field. Following the methodology outlined in Section 2.5, which defines the angle between the magneto-strictive axis and the magnetic field as θ, we conducted tests to assess the magnetic field response at various angles within the range of magnetic field strengths from 0 to 42 mT. For the uniaxial sensors, the relationship between wavelength drift and magnetic field was examined at specific angles (θ=0°, 15°, 30°, 45°, 60°, 75°, and 90°). The data at other angles were then interpolated using cubic spline polynomial interpolation to establish a three-dimensional (3D) mapping curve that relates wavelength drift to angle and magnetic field strength. Figure 8a–c illustrates the resulting spatial mapping curve. The experimental findings indicate strong agreement among the test results obtained from the three uniaxial sensors, affirming the excellent repeatability of our designed sensor.

### 3.3. Vector Magnetic Field Demodulation

The demodulation of vector magnetic field information relies on the correlation between the drift of the center reflection wavelength of the three uniaxial sensors and the intensity and angle of the magnetic field. As depicted in Figure 8d–f, the plane encompassing the center reflection wave peaks exhibits an intersecting curve with the 3D mapping curve. The 2D characteristics of points on this curve correspond to the magnitude and direction of the magnetic field component, hence earning it the designation of magnetic field component curve. The three angles, denoted as $\theta_{x}$, $\theta_{y}$, and $\theta_{z}$, represent the angles between the axis sensors and the measured vector magnetic field direction. The interrelationship among these angles must satisfy the derived Equation (12):

(12) $$\cos{\left(\theta_{x}\right)}^{2}+\cos{\left(\theta_{y}\right)}^{2}+\cos{\left(\theta_{z}\right)}^{2}=1,$$

The 3D vector magnetic field sensor was positioned at the center of a one-dimensional coil in a randomized manner while a stable magnetic field within the range of 3−27 mT was applied. Real-time detection of the reflected wave peaks at the center of the three single-axis sensors facilitated the demodulation of the current vector magnetic field strength. Once the vector magnetic field strength was determined, the pinch angles ($\theta_{x}$, $\theta_{y}$, $\theta_{z}$) and the magnitudes of the component magnetic fields ($H_{x}$, $H_{y}$, $H_{z}$) could be easily calculated based on the magnetic field component curves. For instance, when the magnetic field strength was 25 mT, the central reflection peaks of the three uniaxial sensors were recorded as $peak_{x}$ = 1555.035 nm, $peak_{y}$ = 1555.208 nm, and $peak_{z}$ = 1555.098 nm, respectively. Figure 8d–f illustrates the magnetic field component curves corresponding to the three uniaxial sensors in space.

**Figure 9:**
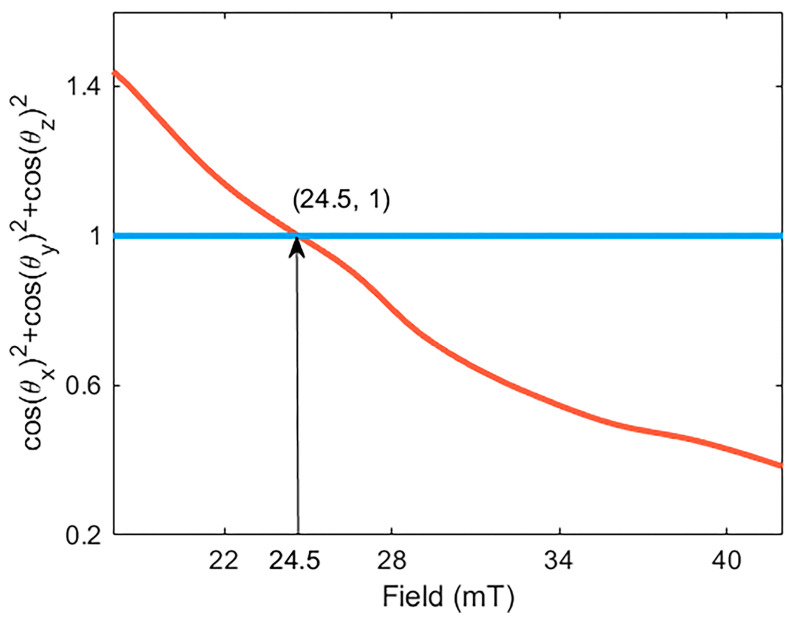
Vector magnetic field demodulation is achieved by intersecting the 3D mapping curve of each single-axis sensor with the plane of the respective central reflection wave peak in space.

The angle between the three uniaxial sensors and the direction of the measured vector magnetic field is determined by Equation (12), and the sum of the residual squares is depicted by the black curve in Figure 9. The magnetic field strength corresponding to the intersection with the straight line y=1 represents the magnitude of the current vector magnetic field, denoted as 24.5 mT. Notably, there exists an error of 0.5 mT between the actual magnetic field strength measured by the Hall probe. This discrepancy can be attributed to the hysteresis effect exhibited by the magneto-strictive material Terfenol-D, which in turn induces hysteresis in the sensor’s response. The presence of hysteresis can detrimentally impact the performance of the sensing system. Furthermore, the nonlinearity of the functional relationship between the magnetic field and the sensor’s wavelength shift, coupled with the influence of magnetic field device accuracy and the fixed position of the fiber-optic sensor, contribute to the inevitable presence of errors between the measured and ideal magnetic field strengths.

**Figure 10:**
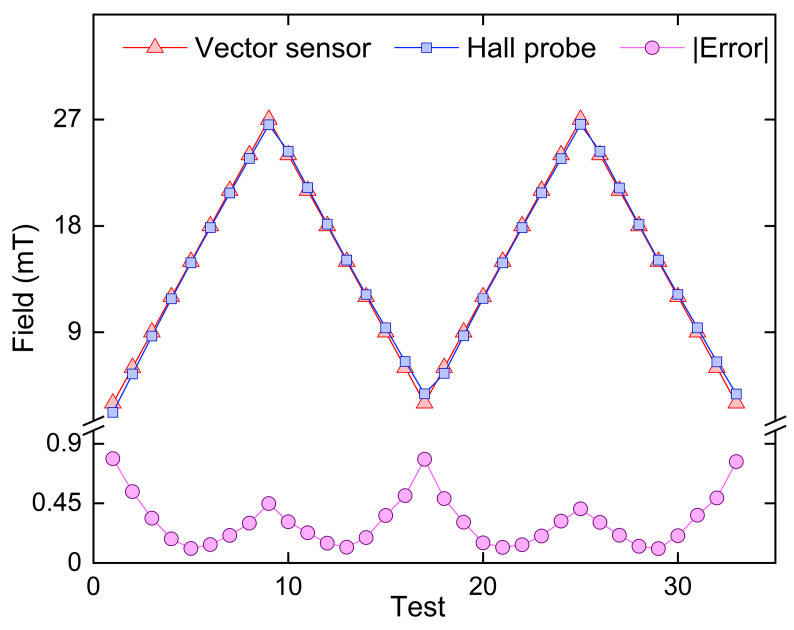
A comparative analysis is conducted between the magnetic field measurements obtained by the Hall probe and those acquired using the optical fiber vector sensor, along with the absolute error in the measurement.

The non-orthogonal error of the 3D vector magnetic field sensor was calibrated and tested multiple times within the magnetic field range of 3−27 mT. The measured magnetic field strengths obtained from the Hall probe were compared with those obtained from the 3D vector fiber-optic magnetic field sensor. The results of these tests are presented in Figure 10. The sensor exhibits a substantial measurement error at both the beginning and end of its linear region, while the error in the middle section is comparatively smaller. This observation highlights the influence of sensor stability on measurement accuracy. In multiple measurements, the maximum error recorded for the sensor is 0.7865 mT. In addition, it should be noted that the application of a magnetic field to the magneto-strictive axes induces stretching and straining in Terfenol-D, resulting in positive magneto-strictive effects for both positive and negative magnetic fields. However, conventional vector sensors face limitations due to the unipolar nature of Terfenol-D, making it challenging to accurately distinguish between positive and negative magnetic fields. In contrast, the vector magnetic field sensor developed in this study enables precise measurements of the vector magnetic field intensity as well as the magnetic field and angle of each uniaxial sensor. This sensor offers a viable solution for the detection and characterization of vector magnetic field information.

## 4. Discussion

The fiber-optic magnetic field sensor presented in this study successfully enables the simultaneous detection of magnetic field and temperature parameters. Through rigorous experimental verification, we have achieved the detection of 3D vector magnetic fields. This subsection discusses the uncertainty and performance comparisons of various fiber-optic magnetic field sensors.

### 4.1. Uncertainty Estimation

In actual testing, sensors can be influenced by various relevant physical quantities, which may introduce bias and errors in magnetic field measurements. Thus, it becomes essential to discuss and account for the uncertainties associated with these relevant physical factors to enhance the confidence of the measurement data. To achieve this, a generalized approach is adopted to assess the uncertainty of different physical covariates using Type A or B assessment methods, which are employed to evaluate the uncertainty of magnetic field-related measurements. In the Type A method, the test system’s data set is non-repeatable, necessitating the statistical analysis of multiple test data sets to determine the associated uncertainty. The standard uncertainty is computed by properly analyzing the experimental standard deviation of the averaging process or mean [22].

To evaluate the Type A uncertainty values, we conducted a series of n=10 tests to analyze the magnetic field response within the linear range of 3−27 mT. The collected test data were then statistically analyzed, and the experimental standard deviation $s\left(H\right)$ was compiled for each magnetic field strength, as shown in Table 1. The standard deviation of the mean was determined as $u\left(\bar{H}\right)=s\left(\bar{H}\right)=s\left(H\right)/\sqrt{n}$. The resulting Type A uncertainty of the mean $u_{1}\left(\bar{H}\right)$ was calculated to be 84.5 nT [23].

The optical sensors proposed in this paper, designed for magnetic field detection in the ocean, may have Type B uncertainties due to various environmental factors present in the ocean environment, such as (1) variations in seawater pressure; (2) fluctuations in seawater temperature; (3) the impact of seawater flow velocity; and (4) resolution limitations of the measuring instrument in the presence of these factors. We plan to design a spherical encapsulated structure based on the principle of a hydrophone to fix the fiber-optic vector magnetic field sensor in the center of the sphere. This structural configuration is instrumental in mitigating the influence of physical factors, such as pressure and water flow, during the actual magnetic field measurements. Furthermore, the double grating cascade of the sensing device, as designed in this paper, exhibits insensitivity to temperature, thereby effectively eliminating the influence of temperature changes on magnetic field measurements. Additionally, we utilized a high-speed grating demodulator (OSI237) with enhanced resolution, capable of achieving precision up to 1 pm. Given the sensor’s high magnetic field sensitivity, the resolution of the designed sensor was calculated in this study. Sensor resolution is typically employed to assess the ability to detect minute changes in the magnetic field and can be evaluated using the following Equation (13) [24]. Where $\Delta \lambda_{min}$ represents the minimum spectral resolution of the high-speed grating demodulator (1 pm), and $S_{H-peak_{a}}$ is the magnetic field sensitivity of the sensor. Based on the calculation, the magnetic field detection resolution of the designed sensor is found to be 87 nT, indicating its capability to respond to magnetic field changes on the order of nanotesla magnitude to a certain extent.

(13) $$R=\frac{\Delta \lambda_{\min}}{S_{H-peak_{a}}},$$

In conclusion, we have taken into account the significance of measurement-related uncertainties in ensuring the reliability of our test data. For the assessment of Type A uncertainty values, a series of repeatability experiments were conducted. The results indicate a Type A uncertainty of mean $u_{1}\left(\bar{H}\right)=84.5\;\mathrm{nT}$, while Type B uncertainty is effectively eliminated through the implementation of the spherical package structure design. The effective degree of freedom $v_{eff}$ of the standard uncertainty is calculated using the Welch–Satterthwaite formula, yielding $v_{eff}=9$ [25]. By applying the student’s t-distribution, a coverage factor k for approximately 95% confidence level and $v_{eff}=9$ is determined to be 2 [26]. Therefore, the expanded uncertainty $U\left(H\right)=k\times u_{1}\left(H\right)$ amounts to 169 nT. The magnetic field sensitivity of the sensor was found to be 11.5±0.17 mT/pm.

### 4.2. Performance Comparison

We provide a comprehensive summary of the fabrication methods and performance characteristics of magnetic field sensors employing various sensing structures. In comparison to the magnetic field sensors documented in Table 2, the dual-parametric magnetic field sensor proposed in this paper achieves higher sensitivity. Additionally, the sensor leverages a multi-wavelength demodulation technique to accurately extract the sensitivity matrix.

In contrast to the magnetic field sensors discussed in previous literature [5,10,11,12,13,27], which are limited to measuring only the magnetic field strength and do not provide information regarding the direction of the magnetic field. This work presents a novel vector magnetic field sensor construction, enabling the measurement of vector magnetic field information. Notably, the proposed sensor incorporates temperature compensation, rendering it insensitive to temperature variations. This compensation significantly reduces the influence of temperature crosstalk on magnetic field detection, ensuring accurate measurement of vector magnetic field information. The studies conducted by Zhan et al. have revealed that Terfenol-D+FBG magnetic field sensors are susceptible to temperature variations, displaying worse stability [13]. Consequently, a thorough examination and experimental verification of the temperature crosstalk effect are essential. Although microstructured fiber-optic sensors introduced in existing literature [6,27] demonstrate high magnetic field sensitivity, the inherent small diameter of the optical microfiber coupler (OMC) results in relatively poor stability. Typically, additional materials are required to encapsulate the sensor, which introduces new challenges. Furthermore, Shao et al. reported vector responses in a 2D plane; their experimental results exhibited unstable characteristics [28]. The vector sensor proposed by Lin et al., which combines magnetic field (MF) and C-type optical fiber (CTF), demonstrates excellent magnetic field sensitivity [13]. However, the sensor’s structural limitations result in suboptimal two-dimensional vector symmetry, with response limited to 0° and 90° angles. In comparison to Terfenol-D+PS-FBG, the Terfenol-D+FBG sensor proposed in this paper not only enhances magnetic field sensitivity and mitigates temperature crosstalk issues but also exhibits superior three-dimensional vector characteristics. Compared with the same type of sensor, the 3D vector magnetic field sensor proposed in this study possesses notable advantages, including high sensitivity, compact size, excellent vector performance, and superior stability. These merits position the sensor as a valuable device for monitoring and measuring magnetic field information in oceanic environments.

**Table 2:** Performance Comparison of Various Optic Fiber Magnetic Field Sensors.

Sensing Structure	Magnetic Field Sensitivity	Measuring Range	Vector Measurement	Sensitivity Matrix Establishing	Ref.
MF+OMCI	0.96 nm/mT	0–5 mT	Negative	Yes	[5]
MF+CTF	2.02 nm/mT	0–12.3 mT	2D	No	[7]
Terfenol-D Films +FBG	0.905 pm/mT	0–50 mT	Negative	No	[10]
Terfenol-D Films +S-FBG	0.7 pm/mT	0–140 mT	Negative	No	[11]
Terfenol-D+FBGs	0.87 pm/mT	8–28 mT	Negative	YES	[13]
Terfenol-D+OMC	0.178 nm/mT	0–40 mT	Negative	No	[27]
Terfenol-D+PS-FBG	0.8 pm/mT	2.4–22.5 mT	2D	No	[28]
**Terfenol-D+FBGs**	**11.5 pm/mT**	**3–27 mT**	**3D**	**Yes**	**This work**

Nevertheless, the vector magnetic field sensor proposed in this paper still requires further refinement. Firstly, although the magnetic field sensitivity has been improved by a factor of 5.7 compared to our previous work [29], there is still room for further enhancement. Secondly, the fixed structure of the proposed vector sensor is susceptible to environmental factors, compromising its stability. To address these limitations and enhance the performance of the vector sensor, we propose incorporating a mechanical sensitization structure [30], which is expected to significantly boost sensor sensitivity. Additionally, a spherical closed package structure will be designed to enhance the stability of the vector sensor. Once the fabrication and testing of a single vector magnetic field sensor are successfully accomplished, the next step involves constructing magnetic field sensing networks to detect the spatial magnetic field and oceanic magnetic field information. Moreover, by cascading gratings, multiple parameters such as temperature, magnetic field, salinity, pressure, etc. can be demodulated. These objectives will drive our future research endeavors.

## 5. Conclusions

In this paper, we present the Terfenol-D+FBG vector magnetic field sensor, which exhibits high sensitivity to both magnetic field and temperature. Through experimental verification, we establish a sensitivity matrix to mitigate the cross-sensitivity between magnetic field and temperature. Importantly, we successfully demodulate the information of the vector magnetic field, including both its intensity and direction. Based on our findings, the following key conclusions can be drawn from this study:

(1) The uniaxial sensor demonstrates reliable magnetic field detection within the range of 3−27 mT at a consistent temperature, exhibiting a magnetic field sensitivity of $S_{H-{peak}_{a}}=11.5\;\mathrm{pm}/\mathrm{mT}$.
(2) Under the condition of magnetic field shielding, the cascaded grating sensors exhibit temperature sensitivities of $S_{T-{peak}_{a}}=13.3\;\mathrm{pm}/\mathrm{mT}$ and $S_{T-{peak}_{b}}=10.5\;\mathrm{pm}/\mathrm{mT}$, respectively, within the temperature range of 14 °C to 38 °C. The implementation of temperature compensation methods effectively mitigates the impact of temperature crosstalk on magnetic field detection.
(3) The sensor exhibits commendable two-dimensional vector characteristics in the X–Y plane, displaying a detection period of 180°. Moreover, it showcases similar characteristic responses within the 0−180° and 180°−360° periods.
(4) By demodulating the relationship between wavelength drift, magnetic field strength, and the angle *θ*, we obtain the vector magnetic field’s strength and direction. The sensor’s stability is rigorously assessed, with a maximum error recorded at 0.7865 mT. The proposed sensor in this study accurately measures the intensity of the vector magnetic field, along with the component magnetic field and angle of each single-axis sensor.

The utilization of FBG and magneto-strictive material in the magnetic field sensor allows for conveniently arrayed sensing. However, the performance of weak magnetic field detection in this type of sensing requires further enhancement. The vector sensor presented in this study offers notable advantages, including its compact size, affordability, excellent vector characteristics, and seamless integration with other optical components. Its application holds significant value for the advancement of marine-based endeavors, such as underwater magnetic field network detection and the monitoring of oceanic magnetic anomalies.

## Figures and Tables

**Table 1:** The magnetic field response was tested 10 times within the linear range of 3–27 mT.

**Hall Probe (mT).**	3	6	9	12	15	18	21	24	27
**Vector Sensor** **1st Test (mT)**	2.2135	5.4623	8.6618	11.8187	14.8913	17.8613	20.792	23.6994	26.5523
**2**	3.6844	6.5077	9.3582	12.1913	15.1187	18.1487	21.228	24.3106	27.4552
**3**	3.7826	5.5144	8.6923	11.8486	14.8824	17.8631	20.7977	23.6851	26.592
**4**	3.7641	6.4912	9.3657	12.2051	15.1082	18.1259	21.2093	24.3064	27.4281
**5**	2.5357	5.9138	8.6832	11.8138	14.998	18.0539	20.9202	24.1191	26.7818
**6**	3.3491	6.1244	8.718	11.9755	14.9891	18.0465	21.0426	24.2345	27.3066
**7**	3.434	5.7369	9.2264	11.9526	15.0293	17.8988	20.8619	24.2756	26.7789
**8**	3.1787	6.1648	9.1364	12.1062	15.0419	17.8857	21.1256	24.0283	27.2829
**9**	3.2577	5.6255	8.872	12.1181	15.0509	17.9995	20.8775	23.7832	26.7692
**10**	3.2431	5.8215	9.3152	11.8747	14.9552	18.1379	21.003	23.7896	27.3863
**Average**	3.2428	5.9363	9.0029	11.9905	15.0065	18.0021	20.9858	24.0232	27.03333
**Experimental standard** **deviation**	0.5818	0.3763	0.3049	0.1535	0.0805	0.1171	0.1619	0.2607	0.3681
**Type A uncertainty**	0.1839	0.1190	0.0964	0.0485	0.0255	0.0370	0.0512	0.0824	0.1164

## Data Availability

The data presented in this study are available on request from the corresponding author.
